# Synthesis and Antibody Binding Studies of Schistosome-Derived Oligo-α-(1-2)-l-Fucosides

**DOI:** 10.3390/molecules26082246

**Published:** 2021-04-13

**Authors:** Michael R. Harvey, Fabrizio Chiodo, Wouter Noest, Cornelis H. Hokke, Gijsbert A. van der Marel, Jeroen D.C. Codée

**Affiliations:** 1 Leiden Institute of Chemistry, Leiden University, Einsteinweg 55, 2333CC Leiden, The Netherlands; m.r.harvey@LIC.leidenuniv.nl (M.R.H.); f.chiodo@amsterdamumc.nl (F.C.); wnoest@gmail.com (W.N.); marel_g@chem.leidenuniv.nl (G.A.v.d.M.); 2 Department of Parasitology, Leiden University Medical Center, 2333ZA Leiden, The Netherlands; c.h.hokke@lumc.nl

**Keywords:** schistosome, glycans, synthesis, gold nano particles, glycan microarray, glycan antigen

## Abstract

Schistosomiasis is caused by blood-dwelling parasitic trematodes of the genus *Schistosoma* and is classified by the WHO as the second most socioeconomically devastating parasitic disease, second only to malaria. *Schistosoma* expresses a complex array of glycans as part of glycoproteins and glycolipids that can be targeted by both the adaptive and the innate part of the immune system. Some of these glycans can be used for diagnostic purposes. A subgroup of schistosome glycans is decorated with unique α-(1-2)-fucosides and it has been shown that these often multi-fucosylated fragments are prime targets for antibodies generated during infection. Since these α-(1-2)-fucosides cannot be obtained in sufficient purity from biological sources, we set out to develop an effective route of synthesis towards α-(1-2)-oligofucosides of varying length. Here we describe the exploration of two different approaches, starting from either end of the fucose chains. The oligosaccharides have been attached to gold nanoparticles and used in an enzyme-linked immunosorbent assay ELISA and a microarray format to probe antibody binding. We show that binding to the oligofucosides of antibodies in sera of infected people depends on the length of the oligofucose chains, with the largest glycans showing most binding.

## 1. Introduction

**Figure 1:**
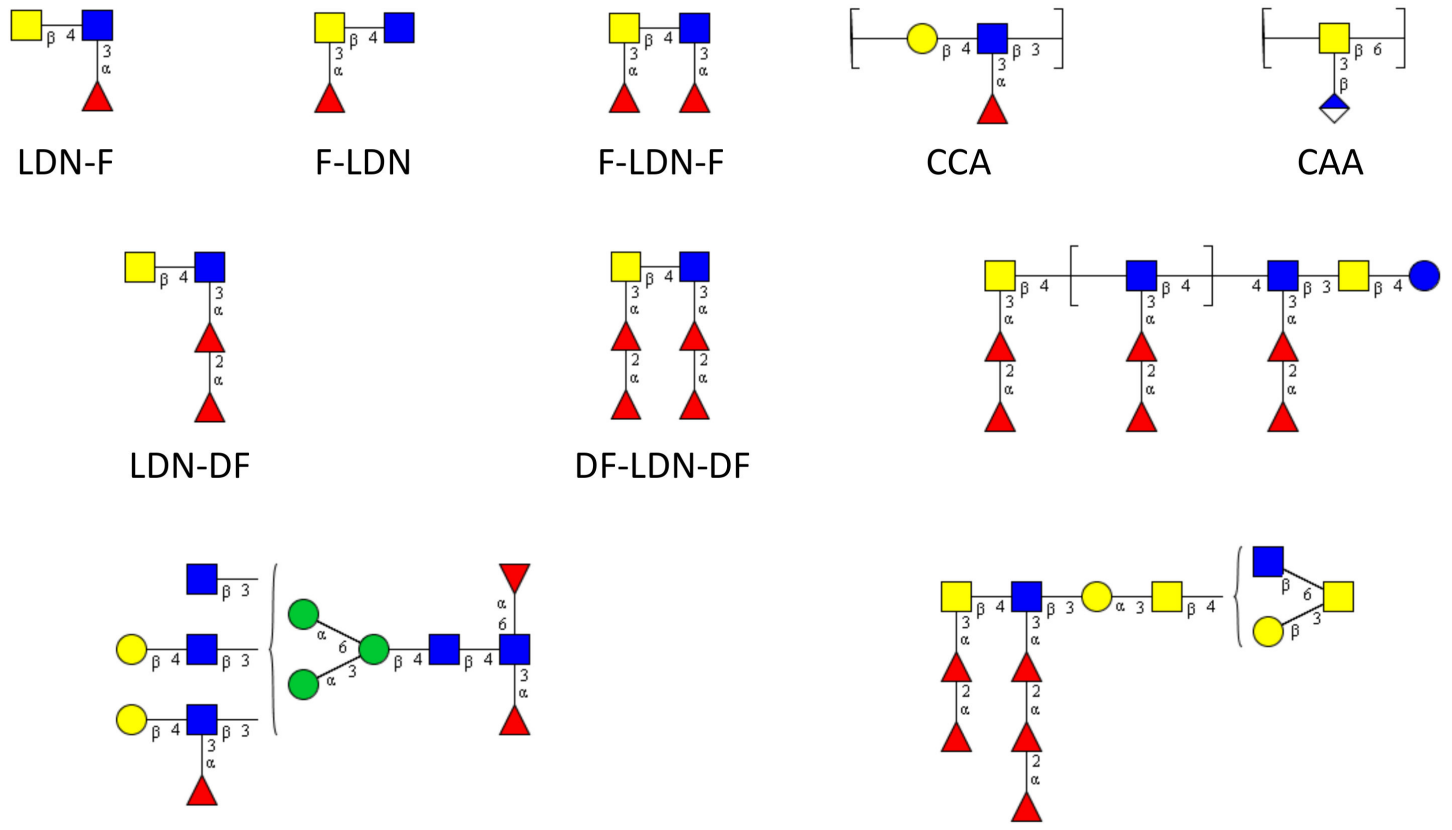
Overview of several observed glycans in *S. mansoni*. Red triangles: l-Fucose, blue square: d-GlcNAc, yellow square: d-GalNAc, blue circle: d-Glucose, yellow circle: d-Galactose, green circle: d-Mannose, and blue/white diamond: d-Glucuronic acid [8,9].

Schistosomiasis, also known as bilharzia, is a major neglected tropical disease caused by parasitic snail-borne trematodes of the genus *Schistosoma* [1]. The World Health Organization (WHO) classified it as the second most socioeconomically devastating parasitic disease, second only to malaria. The number of infections is estimated at 249 million people per year, of which 200,000 ends in a loss of life [2,3]. Schistosomiasis mainly occurs in low- and middle-income countries, especially in areas without access to clean drinking water and inadequate sanitation [4]. *Schistosoma* expresses a complex array of glycans that can be targeted by both the adaptive and the innate part of the immune system [5]. Some of these glycans can be used for diagnostic purposes (see Figure 1) [5,6]. A subgroup of schistosome glycans is decorated with unique α-(1-2)-fucosides [7,8,9,10], and it has been shown that these, often multi-fucosylated, fragments are prime targets for antibodies generated during infection [11,12,13]. The α-(1-2)-fucosides are attached to a backbone of β-(1-4) linked *N*-acetylgalactosamines and/or *N*-acetylglucosamines in the native glycans [6,8,10].

Well-defined α-(1-2)-oligofucosides would be valuable tools to capture specific anti-carbohydrate antibodies or develop conjugate vaccines targeting these unique glycan structures. These α-(1-2)-oligofucosides cannot be obtained in sufficient purity, or quantity from biological sources, as there are no chemical or enzymatic tools available to release these chains from the backbone without jeopardizing the integrity of the fucosyl chains. We, therefore, set out to develop an effective route of synthesis towards α-(1-2)-oligofucosides, of varying length. Previous syntheses have revealed that the construction of the α-(1-2)-fucosyl linkages pose a challenge especially when longer fucose chains are targeted [14,15]. We here describe the exploration of two different approaches, starting from either end of the fucose chains. We have equipped the chains, which vary in length from dimer to tetramer, with a 6-aminohexan-1-ol linker for conjugation purposes. The oligosaccharides have been attached to gold nanoparticles and used in enzyme-linked immunosorbent assay (ELISA) assays and in a microarray format to probe antibody binding. We show that the binding of antibodies in human serum, generated upon *S. mansoni* infection, depends on the length of the oligofucose chains, with the largest glycans showing most binding. The tetra-fucoside that is described here represents an attractive structure to be used in the context of schistosomiasis diagnostics research.

## 2. Results and Discussion

### 2.1. Synthesis of α-(1-2) Oligofucosides

**Scheme 1:**
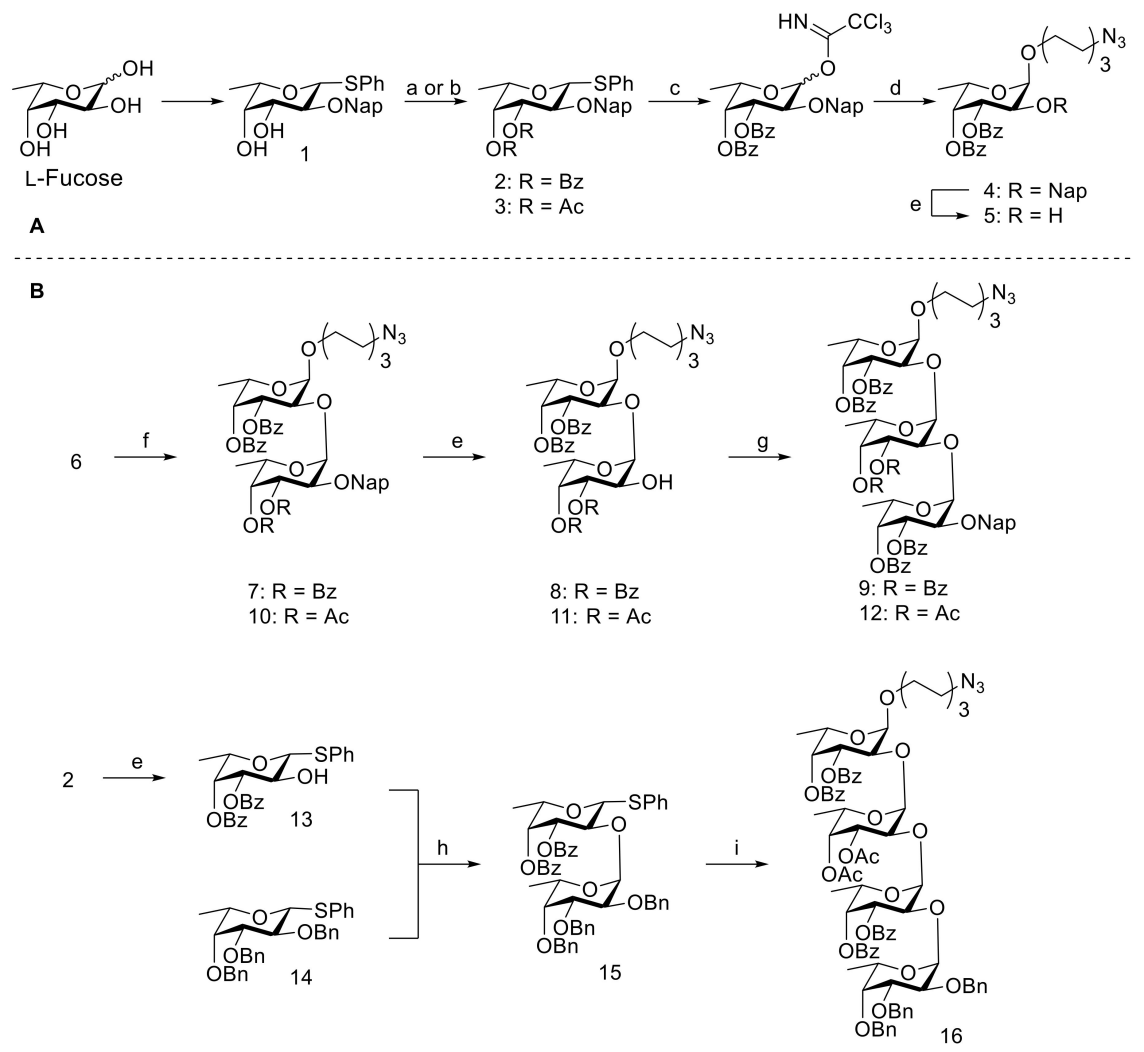
(**A**) Synthesis of building blocks, (**B**) synthesis of α-(1-2) oligofucosides. *Reagents and conditions*: **a**: Ac_2_O, pyr, DCM, 0 °C to RT, 97% **b**: Bz-Cl, DMAP, pyr, DCE, 60 °C, 93%, **c**: (i) NBS, acetone, H_2_O, (ii) CS_2_CO_3_ (cat.), Cl_3_CCN, DCM, 70% (over 2 steps), α/β, 2/1, **d**: 6-azidohexan-1-ol, TMSI, Ph_2_MeP = O, MS (3Å), DCM, 63%, **e**: DDQ, DCM, MeOH, 85% (**6**), 89% (**8**), 87% (**11**), 99% (**13**), **f**: **2** or **3**, NIS, TMSOTf, MS (3Å), DCM, −40 °C to −20 °C, 82% (**7**), 70% (**10**), **g**: **4**, TMSOTf, MS (3Å), DCM, −30 °C, 16% (**9**), 26% (**12**), **h**: IDCP, DCM, 0 °C, 73%, **i**: **11**, NIS, TMSOTf, MS (3Å), DCM, −40 °C to RT, 17%.

We first explored a synthetic route starting from the reducing end as depicted in Scheme 1. The building blocks we used included thioglycoside and imidate donors, equipped with benzoyl or acetyl esters at the C-3 and C-4 hydroxy groups. A naphthyl ether was used as a non-participating protecting group for the alcohol at C-2. Diol **1** was prepared from l-fucose in six steps [16] and while acetylation of both hydroxyl groups in **1** was readily accomplished, benzoylation required rather forcing conditions, using benzoyl chloride in pyridine with 4-(dimethylamino)pyridine (DMAP) at 60 °C, in order to acylate the poorly reactive axial C4-OH. The 6-azidohexan-1-ol linker was installed using a protocol we recently developed for the installation of 1,2-*cis* linkages to relatively reactive (primary) alcohol acceptors. To this end, we transformed the thioglycoside **2** into the corresponding imidate **4** [17,18]. As it was possible to separate both anomers, the following conditions were applied to pure α-imidate, pure β-imidate, and an α/β (2/1) mixture. It was observed that all of these glycosylations resulted in the desired product in a similar yield. Activation of this imidate using (MePh_2_P = O) as an additive in conjunction with TMSI, as originally proposed by Mukaiyama for the activation of anomeric acetates [19,20], and addition of 6-azidohexan-1-ol then provided the spacer equipped fucoside **5** in a 63% yield. Conversion of **5** into acceptor **6** was achieved by cleavage of the naphthyl group with 2,3-dichloro-5,6-dicyano-1,4-benzoquinone (DDQ) in a mixture of dichloromethane and methanol [21].

With the different donor building blocks in hand, the assembly of the oligofucosides was undertaken as shown in Scheme 1b. Acceptor **6** was condensed with benzoyl donor **2** with the N-iodosuccinimide/trimethylsilyltriflate (NIS/TMSOTf) activator couple, giving disaccharide **7** an excellent yield of 82%. The stereoselectivity was >95%, as confirmed by NMR (J_1-2_ = 3.5 Hz and ^1^J_C-1, H-1_ = 170 Hz) [22]. This high selectivity can be attributed long-range participation of the C4-O-benzoyl group [23,24]. Removal of the naphthyl protective group by treatment with DDQ in a mixture of DCM and methanol resulted in acceptor **8** in 89% yield. Dimer acceptor **8** was then condensed with donor **2** using the same NIS/TMSOTf conditions but, in contrast to the successful synthesis of dimer **8**, the yield of trimer **9** was only 7% (Table 1, entry 1), while the stereoselectivity remained excellent (>95%). Switching to imidate donor **4** marginally improved the yield to 16% (Table 1, entry 3). We reasoned that the low yield could be attributed to the bulky nature of the benzoyl groups on the second fucose and the relatively poor accessibility of the disaccharide C2′-OH and we, therefore, explored a disaccharide acceptor bearing the smaller acetyl groups at the C-3′ and C-4′. This disaccharide was obtained from the condensation of acceptor **6** and donor **3**, which gave disaccharide **10** as a 6:1 α/β-mixture, from which the desired α-disaccharide was isolated in 70% yield. Acceptor **11** was obtained after the removal of the naphthyl ether. Several attempts were made to find effective conditions to generate the target trifucoside as summarized in Table 1. Using NIS/TMSOTf in combination with donor **2** led to the predominant formation of anomeric succinimide **17**. The use of imidate donor **4** resulted in an increased yield, but was plagued by the formation of the trichloroacetamide side-product (especially at a higher temperature, Table 1, entry 4). Even when four equivalents of this donor were used we only obtained the target trisaccharide in 26% yield.

Based on the low yields in the assembly of the trisaccharide, the stepwise elongation approach was abandoned and a convergent [2 + 2] approach was explored next to obtain tetrasaccharide **16** (Scheme 1b). To this end, thiofucose acceptor **13** was prepared by DDQ mediated cleavage of the naphthyl ether present on donor **2**. Subsequent chemoselective condensation of perbenzylated armed thiodonor **14 [25]** with disarmed thioacceptor **13** resulted in the formation of disaccharide **15** in a yield of 74% when IDCP was used as the activating agent. No self-condensation of **13** was observed and the stereoselectivity was excellent (7/1, α/β). Condensation of donor **15** with disaccharide acceptor **11** applying the NIS/TMSOTf protocol yielded tetrasaccharide **16** with complete stereoselectivity albeit in a low yield of 17%.

**Scheme 2:**
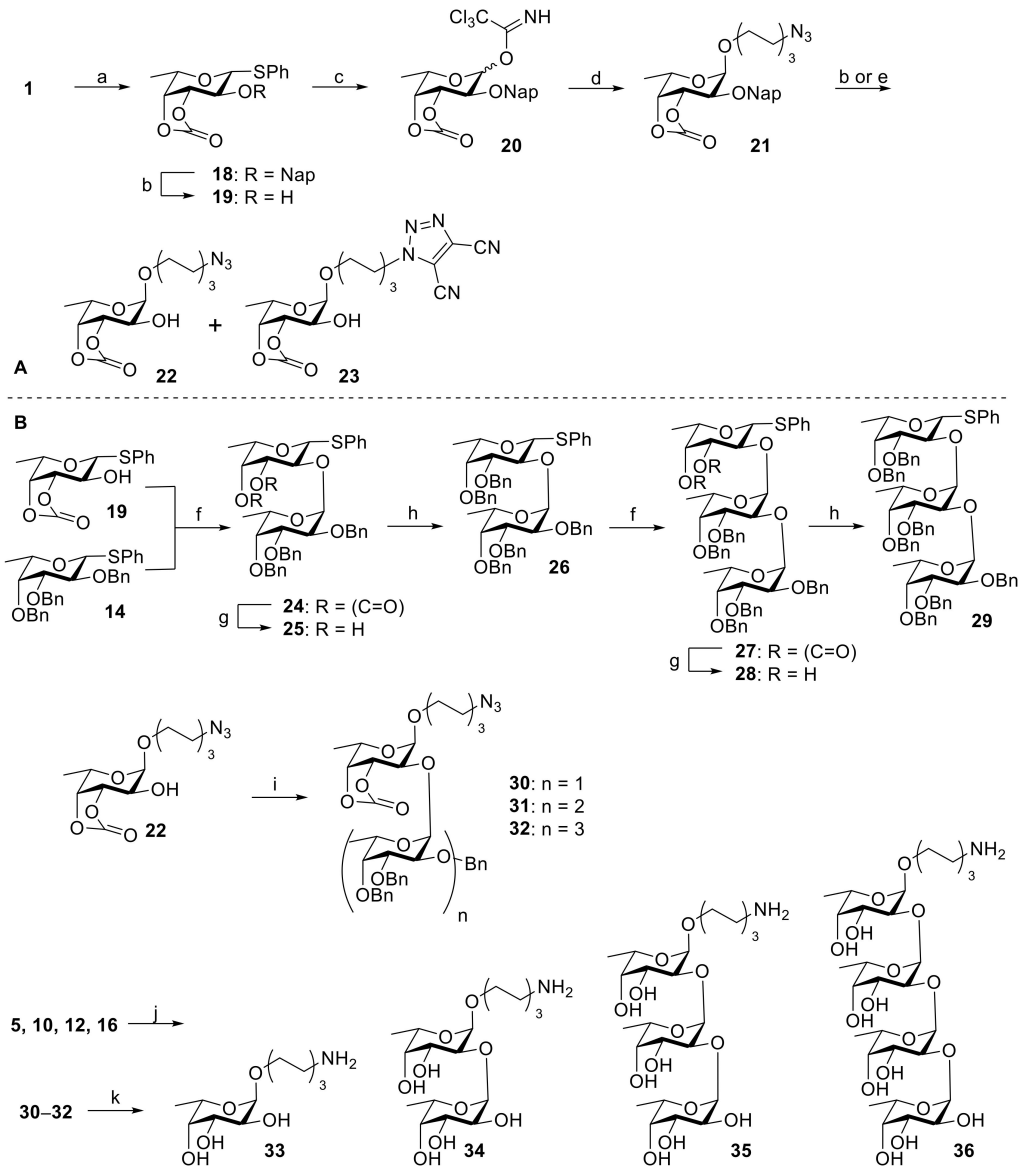
(**A**) Synthesis of building blocks, (**B**) synthesis of α-(1-2) oligofucosides. *Reagents and conditions*: **a**: triphosgene, pyr. DCM, 0 °C, 90%., **b**: DDQ, DCM, MeOH, 78% (**19**), 52% (**22**), 14% (**23**), **c**: (i) NBS, acetone, H_2_O, 81%, (ii) DBU (cat.), Cl_3_CCN, DCM, 77%, **d**: 6-azidohexan-1-ol, TMSI, Ph_2_MeP = O, MS (3Å), DCM, 70%, **e**: HCl (cat.), TES, HFIP, DCM, 65%.**f**: **19**, IDCP, MS (3Å), DCM, 0 °C, 62% (**24**), 78% (**27**) **g**: NaOH, H_2_O, THF, 40 °C, 98% (**25**), 88% (**28**), **h**: BnBr, NaH, TBAI (cat.), DMF, 40 °C, 72% (**26**), 80% (**29**), **i**: **14**, **26** or **29**, IDCP, MS (3Å), DCM, 0 °C, 60% (**30**), 70% (**31**), 61% (**32**), **j**: (i) NaOMe, MeOH/DCM, (ii) Pd/C, H_2_, H_2_O, 31% (**33**), 68% (**34**), 74% (**35**), 74% (**36**) (over two steps), **k**: (i) NaOH, H_2_O, THF, 40 °C, (ii) Pd/C, H_2_, Diox., H_2_O, 40 °C, 48% (**34**), 18% (**35**), 21% (**36**).

Although the desired α-(1-2)-oligofucosides could be synthesized with this method, the low yields during the elongation steps prompted us to try a different approach. As shown in the previous synthetic route, the size of the substituent on the C4-O position can unfavorably influence the yield of the glycosylation reaction. To overcome this, it was envisioned to use the smallest protective group possible, a cyclic carbonate, as the protecting group in the synthesis of the target compounds and we decided to build the α-(1-2)-oligofucosides using chemoselective glycosylations from the non-reducing end as depicted in Scheme 2.

The synthesis of the required carbonate acceptors **19** and **22** is depicted in Scheme 2a and was accomplished by treatment of diol **1** with triphosgene in a mixture of DCM and pyridine to give cyclic carbonate **18** in 90% yield [26]. Next, the naphthyl ether was removed using DDQ in a mixture of DCM and methanol, giving alcohol **19** in 78% yield. Terminal building block **22** was synthesized from **18** in four steps. Initially, the thiophenyl group was hydrolyzed using NBS in wet acetone, followed by imidoylation of the formed hemiacetal [27]. This gave imidate donor **20** in a yield of 62% over two steps. The condensation of 6-azidohexan-1-ol with either the α- or the β-imidate of **20** using the previously described MePh_2_P = O/TMSI protocol gave **21** in 70% yield and excellent selectivity [19]. Next, the naphthyl ether was removed by DDQ in a mixture of DCM and methanol. However, this reaction proceeded extremely sluggish and a significant amount (14%) of byproduct was formed. After careful NMR, IR, and HRMS analysis this side product was determined to be **23**. A similar product has previously been reported by Deng and their coworkers [28]. Due to the incompatibility of DDQ with the azide present on the fucoside, a different method, involving the use of HCl in HFIP with triethylsilane as a scavenger, was applied to remove the naphthyl ether [29]. This method resulted in the successful formation of compound **22** in a yield of 65%.

To attain a chemoselective glycosylation procedure between **14** and **19** (Scheme 2b) several activation methods were compared [30]. Initially, NIS in conjunction with a catalytic amount of TMSOTf was tried but this method resulted in a complex mixture of compounds. Next, a pre-activation protocol using Ph_2_SO, TTBP, and Tf_2_O was employed [31], but this led to a relatively low yield (48%) and poor selectivity (α/β = 3:1). The use of IDCP [32] proved effective to promote the chemoselective glycosylation between armed donor **14** and disarmed acceptor **19** and the desired difucoside was obtained in 81% as a 5:1 α/β-mixture (62% isolated) when the reaction was conducted at room temperature. Lowering the temperature to 0 °C increased the selectivity to 6:1, at the expense of a slightly lower yield (72%). With this glycosylation protocol the desired α-(1-2)-oligofucosides were synthesized.

Chain elongation to provide the oligofucosides requires the conversion of disarmed disaccharide **24** into an armed disaccharide. To this end, the carbonate group on disaccharide **24** was removed using sodium hydroxide at 40 °C. Next, the obtained diol **25** was benzylated and, similar to the just described hydrolysis reaction, the temperature of this reaction had to be raised to 40 °C, as the reaction at room temperature didn’t result in complete benzylation. Armed disaccharide donor **26** was then condensed with acceptor **19** with the IDCP conditions to form trisaccharide **27** with complete α-selectivity in a yield of 78%. Disarmed trisaccharide **27** was converted into armed trisaccharide **29** by saponification and benzylation to give armed trisaccharide **29** in 70% yield. Finally, the linker bearing acceptor **22** was condensed with armed donors **14**, **26,** and **29** using the IDCP protocol giving disaccharide **30**, trisaccharide **31,** and tetrasaccharide **32** in excellent stereoselectivity and a yield of 60%, 70%, and 61%, respectively.

The α-(1-2)-oligofucosides obtained from both the first route (**5**, **10**, **12,** and **16**) and the second route (**30**–**32**) were then deprotected. First, the esters/carbonates were removed using Zemplén conditions for the former series and NaOH in a mixture of THF and water for the latter set of fucosides. While the mono- and disaccharides were readily hydrolyzed at room temperature, it was noted that the tri,- and tetrasaccharides required heating to 50 °C to achieve complete saponification of the ester/carbonate groups. Next, the naphthyl and benzyl ethers were removed by hydrogenolysis with the concomitant reduction of the azide to the amine. The hydrogenation of the disaccharide proceeded smoothly at room temperature, while the tri- **30** and tetrasaccharide **31** required elevated temperatures and longer reaction times for full debenzylation. The desired α-(1-2)-oligofucosides could **33**-**36** were obtained from both synthetic routes.

### 2.2. Gold Nanoparticles Functionalized with α-(1-2) Oligofucosides

The detection of anti-glycan antibodies in serum is relevant for evaluating immunity and may find use in future monitoring of schistosomiasis control and elimination efforts [33]. In order to test if the synthesized glycans can be used as a tool to determine the level of schistosome-directed antibodies in infected people, we used them in a solid phase immune assay (ELISA). The glycans were first immobilized on gold nanoparticles (AuNP’s) in order to create a high ligand density on a high-molecular-weight scaffold [33,34,35]. The particles were then absorbed into the ELISA plates and incubated with murine monoclonal antibodies and human *S. mansoni* infection serum.

**Scheme 3:**
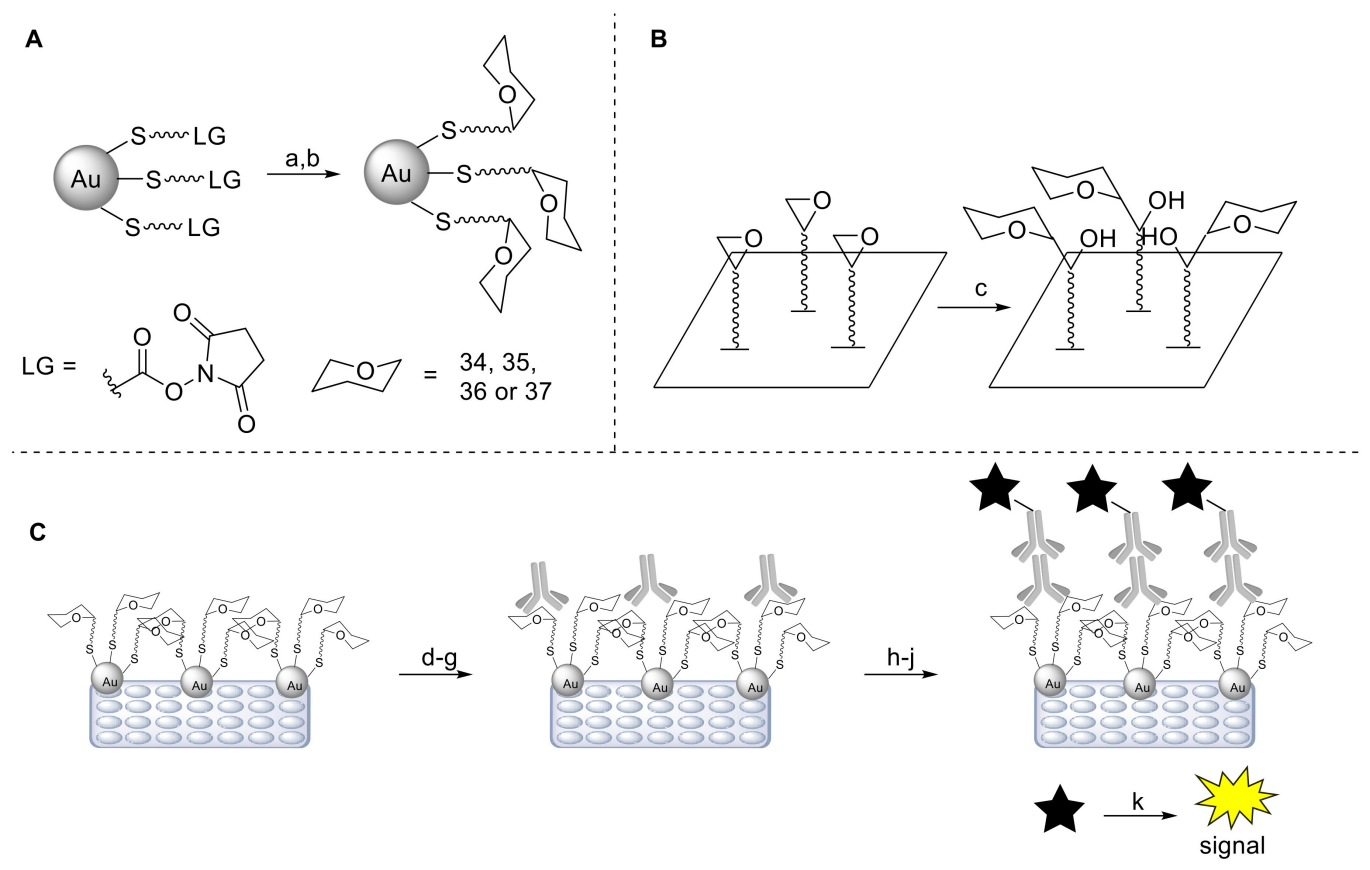
Functionalization of (**A**) gold nanoparticles, (**B)**: glycan array slides. (**C**): ELISA protocol. *Reagents and conditions*: **a**: **33, 34**, **35** or **36**, AuNP, reaction buffer, **b**: quencher solution, **c**: glycan, Et_3_N, H_2_O, **d**: AuNP, coating buffer (50 mM Na_2_CO_3_, pH = 9.6), **e**: washing with PBS buffer, **f**: blocking with 1% BSA, **g**: antibody or human sera, **h**: washing with PBS buffer, **i**: secondary antibody attached to horseradish peroxidase (star), **j**: washing with PBS buffer, **k**: (i) TMB substrate, (ii) H_2_SO_4_.

As depicted in Scheme 3a, the α-(1-2) linked oligofucosides **33**–**36** were covalently linked to pre-formed 5 nm *N*-hydroxysuccinimide activated gold nanoparticles. The so-formed particles were first characterized by screening binding to a set of monoclonal antibodies (mAb’s) that have been shown to recognize glycan motifs present in *S. mansoni.* In the binding studies, soluble egg antigen (SEA), was used as the positive control [36]. Antibodies **I** (258-3E3), **II** (114-5B1-A), and **V** (114-4D12-AA) were used as they have previously been shown to bind di- and tri-fucosylated glucosamine structures, while **III** (291-5D5A) and **IV** (291-4D10-A) can recognize to Lewis X type structures [14,37]. Lastly **VI** (259-2A1) was used as it can bind to GalNAc/LacdiNAc structures.

**Figure 2:**
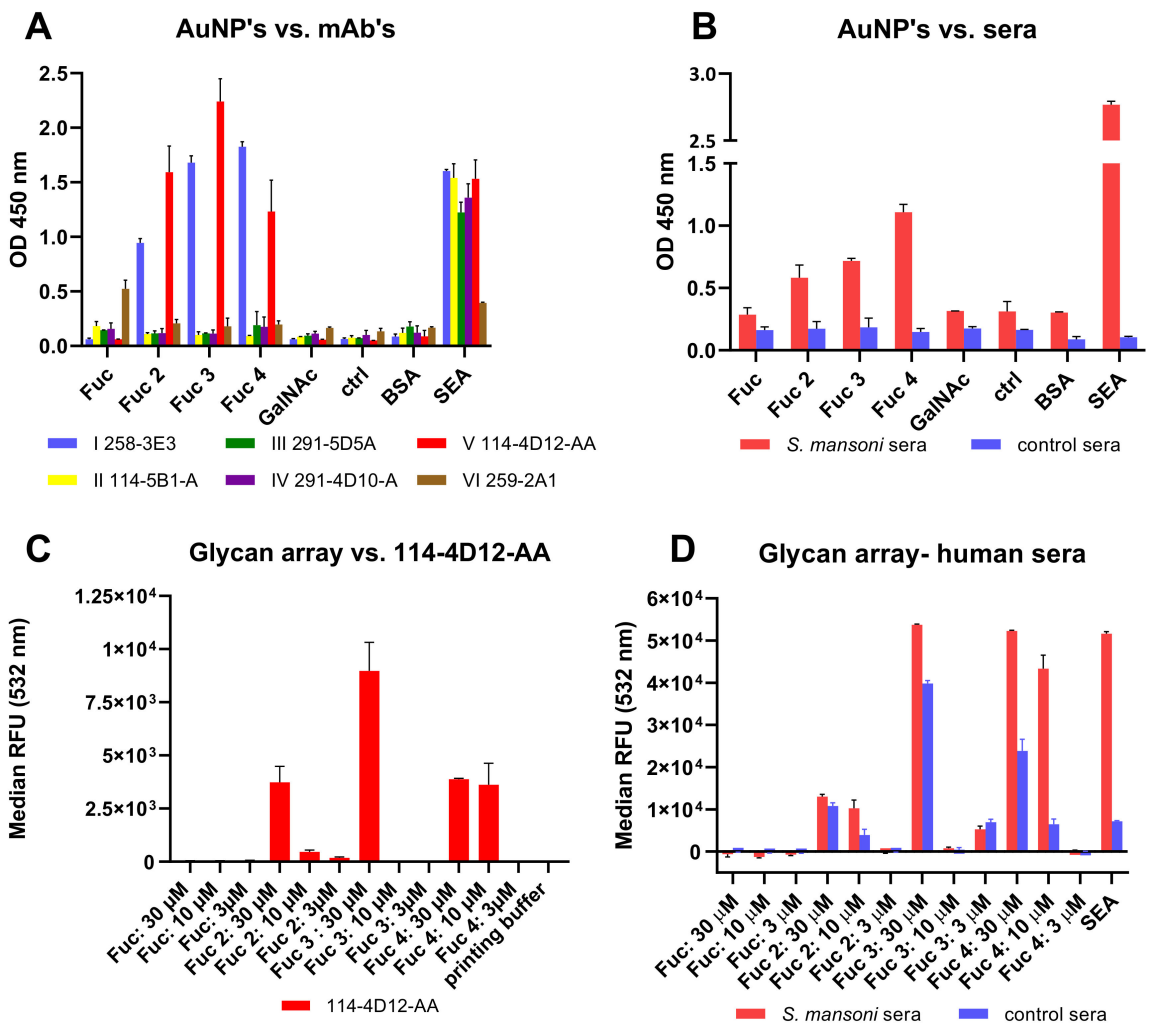
Evaluation of antibody binding to the generated oligofucosides. Bar graph of the results of the ELISA experiment with IgG monoclonal antibodies (**A**) and pooled human sera (**B**) from either infected (red) or control samples (blue). Bar graph of glycan array experiment with mAb 114-4D12-AA (**C**) and with pooled human sera from either infected (red) or control samples (blue) (**D**). AuNP: gold nanoparticle, Fuc **x**: α-(1-2) linked fucosyl chain, where **x** denotes the number of fucoses, GalNAc: 6-aminohexan-1-ol N-acetyl-β-d-galactosamine, ctrl: unfunctionalized AuNP, BSA: bovine serum albumin, SEA: soluble egg antigen, OD: optical density, RFU: relative fluorescence units. Error bars indicate standard deviations of the experiment performed in duplicate (ELISA) or triplicate (glycan array) measurements.

The results of the AuNP ELISA are depicted in Figure 2a, clearly revealing that mAb’s **I** and **V** recognize the α-(1-2) linked fucosyl chains present on the AuNP’s as previously described by Van Roon et al. [14]. The other mAb’s did not show any significant binding, indicating that they do not recognize the α-(1-2) linked oligofucosides, but require other structural elements of schistosome glycan structures, such as LeX or fucosylated LacDiNAc. Interestingly, mAb **II** is able to recognize the di- and tri-fucosylated chains when they are bound to a GlcNAc moiety, but not when the chain is “free”, indicating the backbone itself is also important in binding. The recognition by mAb’s **I** and **V** proved that the synthesized fucosides are accessible to antibodies when bound to the AuNP’s. Next, the AuNP’s were screened against pooled human sera (*n* = 6) from a *S. mansoni*-endemic area in Uganda [38] with pooled non-infected human sera from The Netherlands (*n* = 6) as the control (Figure 2b). The α-(1-2)-linked oligofucosides are recognized by immunoglobulin G (IgG) present in the pooled infected sera, with binding increasing with increasing fucosyl chain length. The unfunctionalized control particles (ctrl) and the AuNP’s functionalized with *N*-acetyl galactosamine (GalNAc) did not show significant binding (O.D. of 0.3) in comparison with the AuNP decorated with oligofucosides in this experiment. It was also shown that the coating with BSA did not influence binding. It is worth noting that the trifucoside is bound preferentially by mAb **V**, however, antibodies in human sera are bound best by the tetrafucoside. In general, AuNP’s functionalized with the longer oligofucosides are preferably bound by IgG in the pooled infection sera, thereby indicating the potential diagnostic use of α-(1-2) fucosides in schistosomiasis.

### 2.3. Evaluation of α-(1-2) Oligofucosides by Glycan Array

To further verify the binding of the α-(1-2)-fucosides, the fragments were evaluated in a glycan microarray set-up (Scheme 3b). Carbohydrate microarrays have become important tools for binding studies of glycans, because they have the advantage that many glycan interactions can be assessed simultaneously while using minimal amounts of sample. The glycans were screened against the monoclonal antibody **V**, the pooled schistosomiasis serum, and the pooled negative control serum, with different glycan printing concentrations (30 μM, 10 μM, and 3 μM). The results of the microarray are shown in Figure 2c,d. The screening against **V** (Figure 2c), shows that the di- and trisaccharide **34** and **35** can be recognized when printed at a concentration of 30 µM. The lower concentrations did not show any significant binding. The interaction of **V** with tetramer **36** appears to be somewhat stronger as binding is also detectable at 10 µM. The binding specificity of this antibody confirms the findings of the AuNP-ELISA experiment. When essaying the array with pooled schistosomiasis serum (Figure 2d), it is again revealed that the longer oligofucosides show stronger binding with IgG, with the tetrafucoside emerging as the strongest binder, corroborating the findings of the ELISA experiments (see the Supplementary Materials)

## 3. Materials and Methods

Glassware used for reactions was oven-dried at 80 °C before use. Anhydrous solvents were prepared by drying them over flame dried activated molecular sieves (3 Å) for at least 24 h before use. Reactions that required anhydrous conditions were co-evaporated with anhydrous toluene and kept under a nitrogen atmosphere. EtOAc and toluene used for silica gel column chromatography were distilled before use, all other chemicals were used as received.

One- and two-dimensional NMR spectra were recorded at 298 K unless stated otherwise on a Bruker AV-300 (300 MHz for ^1^H nuclei and 75 MHz for ^13^C nuclei,), AV-400 (400 MHz for ^1^H nuclei and 101 MHz for ^13^C nuclei), or a Bruker AV-500 (500 MHz for ^1^H nuclei and 126 MHz for ^13^C nuclei (all from Bruker, Billerica, USA). Chemical shifts (δ) are given in ppm relative to tetramethylsilane or the deuterated solvent. IR-spectra was recorded on a Shimadzu FTIT-8300 (Shimadzu, Kyoto, Japan). HRMS spectra were recorded on a Thermo Finnigan LTQ orbitrap mass spectrometer (Thermofischer, Waltham, MA, USA). Unless stated otherwise all reactions were carried out at room temperature and monitored by thin-layer chromatography (TLC). TLC was carried out on Merck aluminum sheets (silica gel 60 F254). TLC analysis was performed by detecting UV adsorption (254 nm) Merck, Kenilworth, IL, USA) where suitable, and spraying the TLC plate with either 20% H_2_SO_4_ in EtOH or a solution of (NH_4_)_6_Mo_7_.4H_2_O (25 g/L), KOH (1 g/L) in water, or a solution of KMnO_4_ (20 g/L) and K_2_CO_3_ (10 g/L) in water or an anisaldehyde solution containing H_2_SO_4_, glacial acetic acid and p-anisaldehyde in absolute EtOH followed by charring the TLC plate at 150 °C. Silica gel column chromatography was performed on silica gel (40–63 µm particle size, 60 Å pore size). Size exclusion chromatography was carried out on Sephadex™ LH-20 gel.

N-hydroxysuccinimide-Activated Gold Nanoparticles and a conjugation kit were purchased from cytodiagnostics Inc © (Burlington, ON, Canada) and used as described in the accompanying manual.

ELISA’s were performed in an ELISA Nunc MaxiSorp^®^ 96-well immunoplate (Thermo Fisher Scientific, Roskilde, Denmark). Coating buffer: 50 mM Na_2_CO_3_, pH = 9.6. Bovine serum albumin (BSA) (lyophilized powder, ≥98%, pH 7, measured by agarose gel electrophoresis) was used. The positive control used for these ELISA was Soluble Egg Antigen (SEA) (1:200 in coating buffer). BSA (1% in PBS) was used as the negative control [39].

The mAbs used in this study were derived from schistosome-infected mice, and their reactivity towards schistosome-derived glycans was determined as described previously by Smit et al. [36].

The glycan array experiments were performed as described by Berni et al. [40].

## 4. Conclusions

In conclusion, we have explored different routes to synthesize oligo-α-(1-2)-fucosides. In order to generate these molecules, two different approaches were evaluated. In the first route, the α-(1-2)-linked oligofucosides were constructed from the reducing end. Although the α-(1-2)-linked di-fucosides (**7**) was synthesized in good yield, the extension to the tri- and tetramer, proved problematic. The second route consisted of synthesizing the α-(1-2) linked oligofucosides from the non-reducing end. In this route, a small carbonate protecting group was employed. Building blocks with this group could effectively be glycosylated and using an armed-disarmed strategy the protected target oligofucosides were readily obtained. The generated fucosides were used to analyze sera of infected people using AuNP-based ELISAs as well as a glycan array. It was shown that the longest fucosyl chains were recognized best, revealing these molecules as potential diagnostic targets for schistosomiasis serology.

## Figures and Tables

**Table 1:**
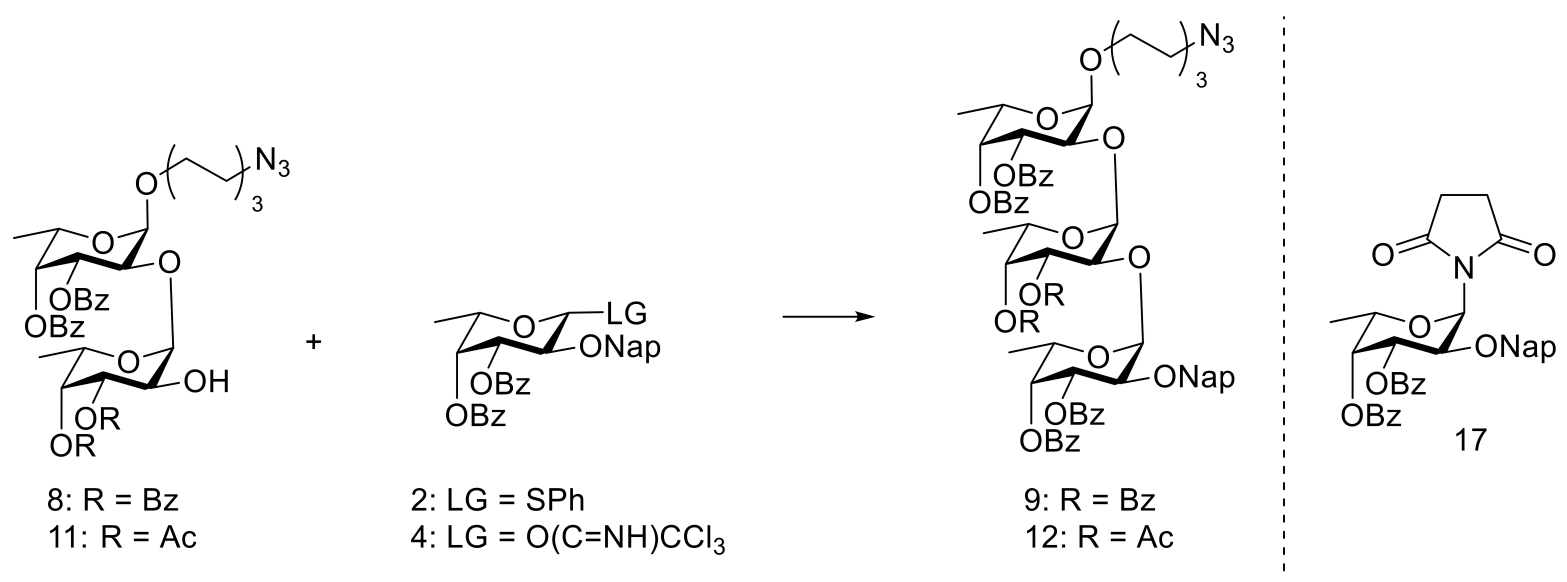
Optimization of chain extension by variation of the C3-O’ and C4-O’ substituents of the acceptor.

Entry	Acceptor	Donor	Activator	T (°C)	Yield (%) ^a^
1	**8**	**2** (1.5 eq.)	NIS (2 eq.), TMSOTf (0.1 eq.)	−40 → 0	7
2	**11**	**2** (1.5 eq.)	NIS (2 eq.), TMSOTf (0.1 eq.)	−40 → 0	10
3	**8**	**4** (1.5 eq.)	TMSOTf (0.1 eq.)	−40 → 0	16
4	**11**	**4** (1.5 eq.)	TMSOTf (0.1 eq.)	−40 → 0	22
5	**11**	**4** (4.0 eq.)	TMSOTf (0.1 eq.)	−30	26

^a^ Isolated yield of the desired trisaccharide.

## Data Availability

The data presented in this study are available in the supporting information.

## Supplementary Materials

The following are available online, Synthesis and characterization of all compounds described in this paper and detailed description of the ELISA experiments.

## Sample Availability

Samples of the compounds are not available from the authors.
